# What do we do now that the long-acting growth hormone is here?

**DOI:** 10.3389/fendo.2022.980979

**Published:** 2022-08-22

**Authors:** Bradley S. Miller

**Affiliations:** Pediatric Endocrinology, University of Minnesota Medical School, Minneapolis, MN, United States

**Keywords:** long-acting growth hormone, pediatric growth hormone deficiency, lonapegsomatropin, somapacitan, somatrogon

## Abstract

In standard 52-week phase III clinical trials, once weekly lonapegsomatropin, somatrogon and somapacitan have been found to yield non-inferior height velocities and similar safety profiles to daily GH (DGH) in children with pediatric growth hormone deficiency (PGHD).

Lonapegsomatropin, a long-acting GH therapy (LAGH), was approved by the United States Food and Drug Administration (FDA) in August 2021 for the treatment of PGHD and has also been approved in other regions of the world. Somatrogon was approved for the treatment of PGHD beginning in some regions beginning in late 2021. Somapacitan was approved by the FDA for the treatment of Adult GHD in August 2020. The phase III clinical trial of somapacitan for the treatment of PGHD has been completed and demonstrated non-inferiority of somapacitan to DGH.

New LAGH products may improve patient adherence, quality of life and clinical outcomes, particularly in patients with poor adherence to daily GH injections in the future. With the availability of new LAGH products, clinicians will need to identify the best candidates for LAGH therapy and understand how to monitor and adjust therapy. Long-term surveillance studies are needed to demonstrate adherence, efficacy, cost-effectiveness and safety of LAGH preparations and to understand how the non-physiological pharmacokinetic and pharmacodynamic profiles following administration of each LAGH product relate to short- and long-term safety and efficacy of LAGH therapy.

## Introduction

This article describes the rationale for using long-acting GH therapy (LAGH), previous attempts at generating LAGH preparations by different pharmaceutical companies, LAGH therapies currently in development or approved around the world, insulin-like growth factor I (IGF-I) monitoring during LAGH therapy, patient selection for LAGH therapy and the future of LAGH.

Daily recombinant human GH (DGH) therapy became available for the treatment of pediatric GHD (PGHD) in 1985 and adult GHD (AGHD) in 1996. However, because of the need for daily injections, individual adherence to GH has been shown to decrease over time with concomitant reductions in height velocity and IGF-I levels in the short term in children and adolescents. In a recent analysis of electronic medical records following initiation of DGH, adherent patients gained an additional 1.8 cm over 1 year compared to non-adherent patients (1). It is likely that reduced adherence to daily injections limits treatment outcomes as evidenced by adult height in children who required GH replacement therapy that are below the mean for the population (2–8). Thus, it has been hypothesized that LAGH products might help mitigate treatment non-adherence and potentially improve long term treatment effects in patients with PGHD.

### Status of long-acting growth hormone products

Nutropin Depot^®^, rhGH released slowly from biodegradable microspheres, was the first LAGH approved for PGHD in 1999, but was removed from the market in 2004 due to marketing and manufacturing issues. Since then, a number of other attempts have been made to develop LAGH products using different approaches to prolong the half-life of the GH molecule (9), including unmodified rhGH in a depot formulation (i.e. Eutropin Plus^®^), pegylated rhGH (i.e. Jintrolong^®^), modification of rhGH to increase albumin binding (i.e. somapacitan, Sogroya^®^), rhGH fusion proteins (i.e. somatrogon, NGENLA^®^) and prodrug releasing unmodified rhGH (i.e. lonapegsomatropin, Skytrofa^®^). Eutropin Plus^®^ (South Korea), Jintrolong^®^ (China), Skytrofa^®^ (lonapegsomatropin; US) and NGENLA^®^ (somatrogon; European Union, Canada, Australia and Japan) are currently approved and available for treatment of PGHD (**Table 1**). Somapacitan (Sogroya^®^; US, EU, Japan) is currently approved for AGHD but not PGHD.

**Table 1:** Long-acting growth hormone characteristics and approval locations.

	Lonapegsomatropin	Somatrogon	Somapacitan
**Mechanism**	Reversible Pegylation	Fusion Protein with hCG CTP (x3)	Acylation increases reversible binding to endogenous Albumin
**Molecular Weight of Active Agent (kDa)**	22	41	23.3
**Approval**	US, EU	EU, Canada, Australia, Japan	US, EU, Japan (AGHD only)

hCG, human chorionic gonadotropin; CTP, c-terminal peptide; kDa, kilodalton; US, United States; EU, European Union; AGHD, adult growth hormone deficiency.

### Lonapegsomatropin

**Figure 1:**
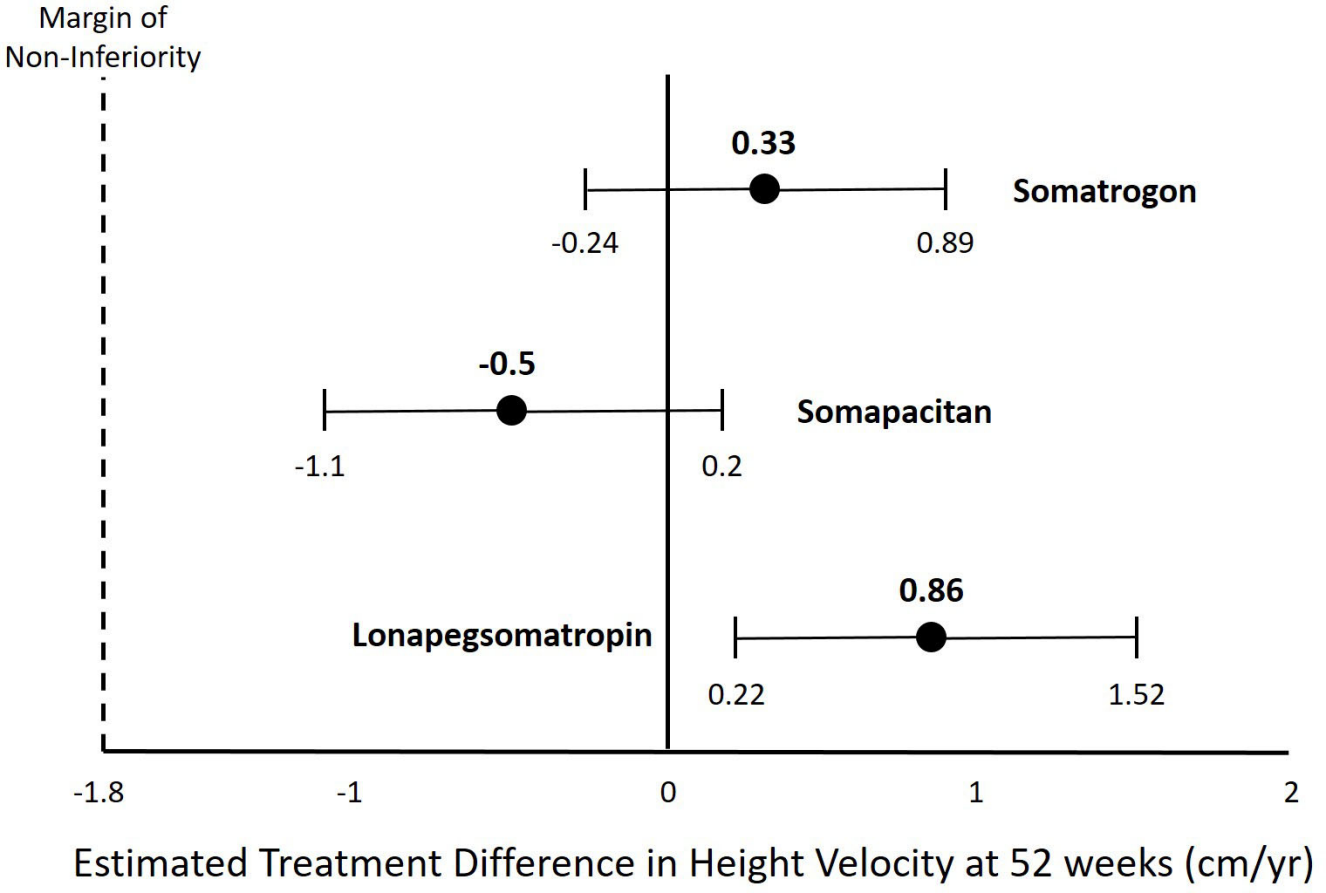
Estimated Treatment Difference in Height Velocity for Different Long-Acting Growth Hormone Products.

Lonapegsomatropin is a prodrug consisting of unmodified GH transiently conjugated to methoxypolyethylene glycol (**Table 1**). This transient chemical modification allows time-release of unmodified GH with a half-life of ~25 hours allowing for once-weekly administration. Clinical trials of lonapegsomatropin have demonstrated positive efficacy results in children (Phase 2 and 3) and adults (Phase 2) with GHD. In the phase III trial of lonapegsomatropin in PGHD, children receiving lonapegsomatropin 0.24 mg/kg once weekly grew 11.2 cm/yr compared to 10.3 cm/yr for children receiving DGH at a dose of 0.24 mg/kg/wk. The estimated treatment difference was 0.86 cm (95% Confidence intervals, 0.22 to 1.50) demonstrating non-inferiority and statistical superiority of lonapegsomatropin compared to DGH (**Table 2**, **Figure 1**) (10). No concerning side effects have been demonstrated with lonapegsomatropin in children or adults.

**Table 2:** Long-acting growth hormone treatment response at 52 weeks.

	Lonapegsomatropin (10)	Somatrogon (11)	Somapacitan (12)
**Dose (mg/kg/wk)**	0.24	0.66	0.16
**Height Velocity LAGH (cm/yr)**	11.2	10.1	11.2
**Height Velocity DGH (cm/yr)** **(0.24 mg/kg/wk)**	10.3	9.8	11.7
**Estimated Treatment Difference (cm/yr), (95% CI)**	0.9 (0.2-1.5)	0.3 (-0.2, 0.9)	-0.5 (-1.1, 0.2)
**Estimated Average** **IGF-I SDS**	+0.72	+0.65	+0.28
**IGF-I SDS DGH**	-0.02	-0.69	+0.10

mg, milligram; kg, kilogram; wk, week; cm, centimeter; yr, year; DGH, daily growth hormone; CI, confidence interval; IGF-I, insulin-like growth factor I; SDS, standard deviation score.

Children receiving lonapegsomatropin in the extension portions of the phase III trials have continued to show efficacy and safety (13–15). After completion of the 12 month pivotal randomized trial comparing lonapegsomatropin to DGH (heiGHt) or completion of the 6 month switch trial (fliGHt), children were able to enroll in the enliGHten extension study. In the most recently available data for the enliGHten trial, at 130 weeks children had reached an average height SDS of -0.64 compared to their midparental target height SDS of -0.39. In the 36 children who completed the trial, they had reached an average height SDS of -0.38 with a difference compared to their mid-parental target height SDS of -0.05 (15).

In the phase III heiGHt trial of lonapegsomatropin in PGHD, children receiving lonapegsomatropin 0.24 mg/kg once weekly had mean IGF-I SDS at week 52 of +0.72 SDS compared to -0.02 SDS in children receiving DGH (**Table 2**). Two children had dose reductions due to asymptomatic elevations of IGF-I (10). In the phase III fliGHt switch trial, the average IGF-I obtained five days after lonapegsomatropin injection was +1.6 SDS. For subjects who had IGF-I values ≤2 SDS at entry into the fliGHt trial (already receiving DGH therapy), 31.2% of children had IGF-I values five days after lonapegsomatropin injection >+2 SDS at 26 weeks (14). In the most recently available data for the enliGHten trial, at 130 weeks children had an average IGF-I value five days after lonapegsomatropin injection of +1.46 SDS (15). Dose reductions of lonapegsomatropin occurred in 29.9% of children in the trial resulting in an average lonapegsomatropin dose of 0.212 mg/kg/wk (13, 15).

### Somatrogon

Somatrogon consists of GH with the addition of three cassettes representing the c-terminal peptide (CTP) of human chorionic gonadotropin resulting in a fusion protein of approximately 41 kDa (**Table 1**). The addition of the CTP cassettes gives somatrogon a prolonged *in vivo* half-life in comparison with native GH allowing for once-weekly administration of somatrogon.

The phase III trial of somatrogon in AGHD (ClinicalTrials.gov Identifier: NCT01909479) was completed in 2016 and failed to meet the primary endpoint (16). However, the phase III trial of somatrogon in PGHD (ClinicalTrials.gov Identifier: NCT02968004) that was completed in 2019 demonstrated non-inferiority of somatrogon to DGH (17, 18). Based upon these results, somatrogon received market authorization for treatment of PGHD in the European Union by the European Medicines Agency in February 2022 (19). Additionally, somatrogon is approved for PGHD in Australia, Canada and Japan and is marketed as NGENLA^®^ (**Table 1**). A Biologics License Application for somatrogon for the treatment of PGHD was submitted to the US Food and Drug Administration (FDA) in 2021 and received a Complete Response Letter in January 2022, but is currently not approved in the US (20). In the phase III trial of somatrogon in PGHD, children receiving somatrogon 0.66 mg/kg once weekly grew 10.1 cm/yr compared to 9.8 cm/yr for children receiving DGH at a dose of 0.24 mg/kg/wk. The estimated treatment difference was 0.33 cm (95% confidence interval, -0.24 to 0.89) demonstrating non-inferiority of somatrogon compared to DGH (**Table 2**, **Figure 1**). No concerning side effects have been demonstrated with somatrogon in children or adults. Anti-drug antibodies have been reported in a significant number of children receiving somatrogon. However, there has not been any demonstration that the reported anti-drug antibodies have been neutralizing or had a negative impact on the growth of children receiving somatrogon. In a report of long-term growth with up to 4 years somatrogon therapy in PGHD from the extension portions of the phase II and III trials, the achieved height velocities and height z-scores were similar or slightly better than expected compared to historical controls from the Pfizer registry KIGS (21).

In the phase III trial of somatrogon in PGHD, children receiving somatrogon 0.66 mg/kg once weekly had mean IGF-I SDS at week 52 of +0.65 SDS compared to -0.69 SDS in children receiving DGH (**Table 2**) (11). There are no data available yet for IGF-I values in children treated with somatrogon for longer periods.

### Somapacitan

Somapacitan consists of GH with one amino acid substitution in an area not involved in GH receptor binding (**Table 1**). An acyl linker that functions as an albumin binding moiety is covalently attached to the modified amino acid. The albumin binding moiety reversibly binds to endogenous albumin gives somapacitan a prolonged *in vivo* half-life in comparison with native GH allowing for once-weekly administration of somapacitan.

Sogroya^®^ (somapacitan; US, Europe, Japan) was approved by the FDA for treatment of AGHD in August 2020, but is yet to be commercially available in the US. It is available in the EU and Japan (**Table 1**). The somapacitan phase III trial in PGHD (ClinicalTrials.gov Identifier: NCT03811535) was completed in 2021 and the results were reported recently, but are not yet published in the peer-reviewed literature (22, 23). In the phase III trial of somapacitan in PGHD, children receiving somapacitan 0.16 mg/kg once weekly grew 11.2 cm/yr compared to 11.7 cm/yr for children receiving DGH at a dose of 0.24 mg/kg/wk. The estimated treatment difference was -0.5 cm (95% confidence interval, -1.1 to 0.2) demonstrating non-inferiority of somapacitan compared to DGH (**Table 2**, **Figure 1**) (12). In a report of long-term growth with up to 4 years somapacitan therapy in PGHD from the extension portions of the phase II trial (REAL3), there was a mean height SDS change from baseline of 2.85 SDS for those who received somapacitan throughout and 2.28 SDS for those who received DGH for two years before switching to weekly somapacitan (24). With a baseline height SDS for the somapacitan groups of approximately -3.8 SDS, the height SDS after 4 years somapacitan therapy would be approximately -0.95 SDS. With a baseline height SDS of -3.4 for the group who received DGH before switching to somapacitan the height SDS after 4 years somapacitan therapy would be -1.12 SDS (25). No concerning side effects have been demonstrated with somapacitan in children or adults.

In the phase III trial of somapacitan in PGHD, children receiving somapacitan 0.16 mg/kg once weekly had mean IGF-I SDS at week 52 of +0.28 SDS compared to +0.10 SDS in children receiving DGH (**Table 2**) (12). In the extension studies of somapacitan, mean IGF-I SDS at year 4 was +1.29 SDS for those who received somapacitan throughout and +0.94 SDS for those who received DGH for two years before switching to weekly somapacitan (24).

### IGF-I monitoring

The pharmacodynamics of the different LAGH products have been measured using IGF-I as the biomarker. Based upon the pharmacodynamic models, the peak IGF-I levels occur between 2 and 3 days and the average IGF-I level occurs between four and six days. Using IGF-I data from the phase 2 and phase 3 clinical trials in children, pharmacodynamic models have been developed to estimate the average IGF-I concentrations from a single serum sample obtained at any time after an injection of LAGH at study state (26–28). It is necessary that the timing of the injection and the timing of collection of the serum sample are known in order to calculate the estimated average IGF-I level. Therefore, if a convenience sample is obtained four to five days after the injection, it is a reasonable estimate of the average IGF-I. If the sample is collected at any other time following the injection, an IGF-I calculator can be used to calculate the estimated average IGF-I. An IGF-I calculator is available for lonapegsomatropin (27). The shape of the pharmacodynamic curve should be identical regardless of the method of IGF-I assay. Therefore, the IGF-I calculator could be very useful regardless of the type of IGF-I assay used. However, these calculators and pharmacodynamic models need to be evaluated further in a broader population of children with GHD, including pubertal children and transition patients. Additionally, since IGF-I values are not normally distributed, the SDS values may vary by age and assay (29). For this reason, further validation of these models with different IGF-I assays is needed.

### Dose adjustment

LAGH has been studied in clinical trials using a weight-based dosing paradigm (11, 12, 15). In this dosing paradigm, similar to DGH, the dose of LAGH was adjusted for the weight of the child at specified clinical research visits. In the published data, the only other dose adjustments occurred were due to elevated IGF-I levels or adverse events. Therefore, there is little information available to guide the clinician on how to adjust the dose of the different LAGH products. In clinical practice, adjustment of DGH dosing has been based upon weight or body surface area, height velocity and/or IGF-I levels. The use of IGF-I levels to guide dose adjustment of DGH therapy has been recommended for both safety and short-term efficacy purposes. From a safety perspective, it has been recommended that GH therapy increases IGF-I levels to rise into the normal range (i.e. ≤ +2 SDS) (30, 31). From an efficacy perspective, targeting an IGF-I in the upper part of the normal range (+1 to +2 SDS) has been suggested in order to improve short-term efficacy (32). However, long-term efficacy of this approach has not been demonstrated. Weight-based dosing of DGH has been shown to achieve an IGF-I level close to 0 SDS depending upon the dose used (33).

As described earlier, the pharmacodynamic profiles of IGF-I levels following an injection of the different LAGH products show a significant increase of IGF-I from baseline to peak and with return to baseline before the next injection (11, 12, 15). It is likely that the efficacy of LAGH will correlate with the average IGF-I level achieved (32). Therefore, it will be important to be able to estimate an average IGF-I from serum samples collected at random clinic visits. Using the IGF-I calculator to estimate average IGF-I values from these samples may help guide dose adjustment of LAGH (11, 12, 15). Lonapegsomatropin, somatrogon and somapacitan have been shown to have a linear IGF-I dose response during clinical trials suggesting that predictable changes in average IGF-I levels can be achieved with adjustments in dose of these LAGH molecules (15, 28, 34, 35).

There have been concerns raised that short-term elevations of GH and IGF-I during LAGH therapy may be associated with short- and long-term adverse events (29, 31). The GH Research Society consensus guidelines suggested that the goal of LAGH therapy is to maintain IGF-I levels within the age-appropriate range for the majority of the treatment period, as IGF-I levels maintained within such age-appropriate range correlates with safety of treatment (31). However, peak IGF-I levels, using the pharmacodynamic model, may be able to be estimated for future analysis of their relationship to safety and efficacy. Clinicians interested in measuring peak IGF-I levels following LAGH administration at steady-state could obtain IGF-I measurements between 2 and 3 days after an injection (26–28).

### Patient selection for LAGH

When selecting children with GHD for treatment with LAGH, providers may consider a number of different characteristics known to negatively impact adherence. Potential candidates for LAGH include individuals with poor adherence, particularly teenagers, young children expected to be on therapy for many years, children with needle phobia, children transitioning to self-injection and patients on multiple other medications, particularly injectable medications like insulin. Good candidates will likely be a highly motivated subset of this list of potential candidates. The prescriber needs to recognize that children with poor adherence with DGH may still have poor adherence with LAGH. Based upon the short-term efficacy and safety data, providers are also likely to start LAGH in naïve children.

Although lonapegsomatropin is approved for PGHD down to 1 year of age and somatrogon is approved for PGHD down to 3 years of age, children with severe GHD associated with hypoglycemia may not be good candidates for LAGH products since they may be at increased risk of hypoglycemia at trough GH levels occurring in the day or two prior to each injection. Since hypoglycemia can occur in children above 3 years of age in isolated PGHD or PGHD associated with multiple pituitary hormone deficiencies, it will be important for providers to recognize this potential risk when considering LAGH therapy. Glucose measurements have been collected in children with PGHD during clinical trials of lonapegsomatropin, somatrogon and somapacitan without any reports of hypoglycemia. However, children with hypoglycemia associated with PGHD would not be naïve at the ages recruited into those clinical trials. Therefore, the occurrence of hypoglycemia in PGHD needs to be studied carefully and may warrant a different dosing paradigm of LAGH products, such as twice weekly injections instead of weekly.

Cancer survivors with PGHD are a group of children who warrant careful thought when considering LAGH therapy. DGH has not been shown to cause recurrent neoplasms, but concern about a small increased risk for subsequent neoplasms overall in pediatric cancer survivors remains (36). Therefore, theoretical concerns about transient elevations of GH and IGF-I that occur with each LAGH dose may lead providers to hesitate when considering LAGH therapy in cancer survivors with PGHD (37). Alternatively, providers may select an LAGH product that has a more flat IGF-I profile with fewer IGF-I excursions above +2 or +3 SDS, a lower LAGH dose or both. If clinicians diagnose PGHD early in cancer survivors, GH replacement therapy may be initiated before the height percentile declines below the normal range, and maintenance of a normal growth rate is sufficient. Thus, use of a higher GH dose to achieve catch-up growth would not be necessary. As more safety and efficacy data for LAGH emerges and as experience with LAGH products grows, it is possible that LAGH may potentially replace DGH in the treatment of PGHD.

### Early experience with LAGH

Since the approval of Skytrofa^®^ (lonapegsomatropin) for PGHD in the US in August 2021, numerous pediatric endocrinologists have begun to prescribe lonapegsomatropin in children. In my personal experience, some children and their families have been reluctant to start lonapegsomatropin instead of DGH or to switch from DGH to lonapegsomatropin. In my practice, the most common reason for families to prefer not to switch to lonapegsomatropin was due to concerns about insurance coverage. In addition to concerns about whether insurance would cover the new product, families were concerned that seeking approval of lonapegsomatropin could lead to a lack of continued approval or renewal of their currently approved DGH. Although this perception may be unique to the US insurance market, it was a powerful deterrent for many families to consider switching to LAGH. It is likely that concern regarding insurance coverage will improve as contracts with insurance providers are developed. FDA approval of other LAGH products may also improve insurance coverage. If adherence and outcomes are demonstrated to be superior in children receiving LAGH, this should also improve insurance coverage of these products. Finally, some children, their families and their providers may be reluctant to transition to LAGH products due to comfort and good growth outcomes with DGH, availability of decades of safety and efficacy data for DGH and fear of change. The cost and cost-effectiveness of LAGH products are also likely to impact treatment decisions. Collection of longitudinal safety and efficacy data for LAGH should help providers provide appropriate reassurance for families interested in treatment with LAGH in the future.

### Future directions

The LAGH products in development have been studied primarily in PGHD. Studies to demonstrate safety and efficacy of LAGH for children with other growth disorders are also needed. A phase II study of Eutropin Plus^®^ (LB03002) in idiopathic short stature (ISS) in South Korea showed evidence of non-inferiority of two doses compared to DGH at 0.37 mg/kg/wk (38). A phase II study of 0.16, 0.20 and 0.24 mg/kg/wk somapacitan (REAL5) in short children born Small for Gestational Age (SGA) demonstrated improved height velocity to 8.9, 11.1 and 11.2 cm/yr, respectively. Children with SGA receiving 0.35 and 0.67 mg/kg/wk DGH demonstrated a height velocity of 10.5 and 11.9 cm/yr, respectively. A phase III clinical trial of somapacitan in short children with SGA, Turner Syndrome, Noonan Syndrome and Idiopathic Short Stature (REAL8) is estimated to start in July 2022 (39).

Although a transition from daily injections to once weekly injections has been shown to improve adherence in other treatment areas, it has not yet been demonstrated in children receiving LAGH products (40–43). It will be important to evaluate adherence through standard methodologies including pharmacy refill data. Novel approaches for capturing adherence such as the Bluetooth capability of the electronic injection device for lonapegsomatropin, if approved, will provide additional information to correlate adherence with treatment outcomes. Although LAGH preparations are being evaluated through a regulatory process that requires demonstration of non-inferiority to DGH injections, it is likely that these compounds will result in improved long-term efficacy as well as convenience for patients and their caregivers. This improvement in outcomes will likely be due to the underestimated impact of reduced adherence and persistence with GH therapy. The data from extension studies of lonapegsomatropin, somatrogon and somapacitan demonstrate the potential for LAGH to close the efficacy gap seen with DGH therapy. Long-term studies, including real world evidence, are still needed to demonstrate these benefits as they are crucial in determining the cost-effectiveness and safety of LAGH preparations.

The long-term safety of LAGH products requires further study as they do not mimic the physiologic profile of endogenous GH secretion or the non-physiologic profile of DGH for which we have extensive safety data. It remains to be seen whether this difference in pharmacokinetic and pharmacodynamic profile will exert a positive or negative impact on short- and long-term safety and efficacy. Following the approval of rhGH in 1985, numerous phase 4 post marketing surveillance registries have collected safety and efficacy data for DGH therapy (44). These studies accumulated more than 600,000 patient years of safety and efficacy data and helped our community learn about common and rare side effects of DGH therapy. One of the challenges of these studies was that children were no longer followed after DGH therapy was discontinued, and were therefore lost to follow-up. In order to collect important data regarding linear growth and metabolic outcomes in children receiving LAGH preparations, including lonapegsomatropin, it will be crucial to perform similar phase IV post marketing surveillance studies. However, in order to avoid losing the patients when they complete therapy or transition to another GH product and to capture patient reported outcomes, it is imperative to develop studies utilizing LAGH therapy that follow children long-term well into their adulthood.

Efforts to develop an independent international study to achieve these outcomes is currently underway (GloBE-Reg LAGH (https://globe-reg.net/)) spearheaded by a consortium of pediatric endocrinology societies. These efforts require support from the manufacturers of DGH and LAGH preparations, as well as from the pediatric endocrinology community. Pfizer has also begun a registry (PROGRES, Pfizer Registry of Outcomes in Growth hormone RESearch) to collect safety and efficacy data in children receiving somatrogon and DGH (45). If registries developed by manufacturers of LAGH products can interact with each other and the GloBE-Reg LAGH study to share data that will increase the power to identify important outcomes. LAGH phase IV registry studies may be useful in validating IGF-I pharmacodynamic models for each LAGH product and in determining the relationship between estimated peak and estimated average IGF-I levels to short- and long-term safety and efficacy of LAGH therapy.

## Conclusion

Numerous LAGH preparations have been or are currently being developed, but they each have their unique molecular characteristics and clinical efficacies (9). In standard 52 week phase III clinical trials, once weekly lonapegsomatropin, somatrogon and somapacitan have been found to yield non-inferior height velocities in children with PGHD with safety profiles comparable to DGH. In longer term extension studies, once weekly lonapegsomatropin, somatrogon and somapacitan have been found to have sustained efficacy in children with PGHD. Thus, LAGH preparations have the potential to close the efficacy gap in DGH by reaching a near adult height appropriate for the mid-parental target height and the population. However, it remains to be seen whether these effects can be replicated in real world use of LAGH. LAGH may improve patient adherence, quality of life and clinical outcomes, particularly in patients with poor adherence to DGH injections. Long-term surveillance studies are needed to demonstrate adherence, efficacy, cost-effectiveness and safety of LAGH preparations and to understand the relationship between estimated peak and estimated average IGF-I levels at steady state to short- and long-term safety and efficacy of LAGH therapy.

## Author contributions

The author confirms being the sole contributor of this work and has approved it for publication.

## Conflict of interest

Dr. Miller is a consultant for AbbVie, Ascendis Pharma, BioMarin, Bristol Myers Squibb, EMD Serono, Endo Pharmaceuticals, Novo Nordisk, Orchard Therapeutics, Pfizer, Tolmar and Vertice and has received research support from Alexion, AbbVie, Aeterna Zentaris, Amgen, Amicus, Lumos Pharma, Lysogene, Novo Nordisk, OPKO Health Pfizer, Prevail Therapeutics and Sangamo Therapeutics.

## Publisher’s note

All claims expressed in this article are solely those of the authors and do not necessarily represent those of their affiliated organizations, or those of the publisher, the editors and the reviewers. Any product that may be evaluated in this article, or claim that may be made by its manufacturer, is not guaranteed or endorsed by the publisher.
